# Effect of surfactant administration on outcomes of adult patients in acute respiratory distress syndrome: a meta-analysis of randomized controlled trials

**DOI:** 10.1186/s12890-018-0761-y

**Published:** 2019-01-09

**Authors:** Shan-Shan Meng, Wei Chang, Zhong-Hua Lu, Jian-Feng Xie, Hai-Bo Qiu, Yi Yang, Feng-Mei Guo

**Affiliations:** Department of Critical Care Medicine, Zhongda Hospital, School of Medicine, Southeast University, No.87, Dingjiaqiao Road, Gulou District, Nanjing, 210009 China

**Keywords:** Acute respiratory distress syndrome, Adult, Surfactant administration, Mortality, PaO_2_/FiO_2_, Oxygenation

## Abstract

**Introduction:**

Surfactant is usually deficiency in adult acute respiratory distress syndrome(ARDS) patient**s** and surfactant administration may be a useful therapy. The aim of this study was to perform a meta-analysis of the effect of surfactant administration on outcomes of adult patients with acute respiratory distress syndrome.

**Methods:**

PubMed, EMBASE, Medline, Cochrane database, Elsevier, Web of Science and http://clinicaltrials.gov were searched and investigated until December 2017. Randomized controlled trials(RCTs) comparing surfactant administration with general therapy in adult patients with ARDS were enrolled. The primary outcome was mortality (7–10-day, 28–30-day and 90–180-day). Secondary outcome included oxygenation (PaO_2_/FiO_2_ ratio). Demographic variables, surfactant administration, and outcomes were retrieved. Sensitivity analyses were used to evaluate the impact of study quality issues on the overall effect. Funnel plot inspection, Egger’s and Begger’s test were applied to investigate the publication bias. Internal validity was assessed with the risk of bias tool. Random errors were evaluated with trial sequential analysis(TSA). Quality levels were assessed by Grading of Recommendations Assessment, Development, and Evaluation methodology(GRADE).

**Results:**

Eleven RCTs with 3038 patients were identified. Surfactant administration could not improve mortality of adult patients [Risk ratio (RR) (95%CI)) = 1.02(0.93–1.12), *p* = 0.65]. Subgroup analysis revealed no difference of 7–10-day mortality [RR(95%CI)) = 0.89(0.54–1.49), *p* = 0.66], 28–30-day mortality[RR(95%CI) = 1.00(0.89–1.12), *p* = 0.98] and 90–180-day mortality [RR(95%CI) = 1.11(0.94–1.32), *p* = 0.22] between surfactant group and control group. The change of the PaO_2_/FiO_2_ ratio in adult ARDS patients had no difference [MD(95%CI) = 0.06(− 0.12–0.24), *p* = 0.5] after surfactant administration. Finally, TSA and GRADE indicated lack of firm evidence for a beneficial effect.

**Conclusions:**

Surfactant administration has not been shown to improve mortality and improve oxygenation for adult ARDS patients. Large rigorous randomized trials are needed to explore the effect of surfactant to adult ARDS patients.

**Electronic supplementary material:**

The online version of this article (10.1186/s12890-018-0761-y) contains supplementary material, which is available to authorized users.

## Background

Acute respiratory distress syndrome (ARDS) is characterized with diffuse lesions of pulmonary endothelial and alveolar epithelium cells, resulting in alveolar and interstitial tissue flooding and edema, reduced lung compliance, imbalanced lung ventilation flow ratio, decreased lung volume, and refractory dyspnea [1]. In recent years, mechanical ventilation is regarded as the main therapeutic management for ARDS. The mortality rate of ARDS is decreasing whereas as high as 30–50% with the continuous optimization of mechanical ventilation strategy [2]. Given the high mortality rate of ARDS patients, other effective therapies are still needed.

In the early stage of ARDS, surfactant deficiency and dysfunction may be a result of the loss in alveolar epithelium, which impairs surface-tension-lowering and results in bad gas exchange and lung injury. Pulmonary surfactant is produced by type II pulmonary epithelial cells and mainly consists of three components: phospholipids, neutral fat and surfactant-specific proteins (including SP-A, SP-B and SP-C et al). Surfactant can reduce alveolar surface tension, thereby preventing alveolar collapse. Furthermore, pulmonary surfactant can enhance phagocytes function and maintain immune response in the patients of ARDS [3]. The mechanisms of action for surfactant in ARDS were detailed in Table 1. In view of above properties, administration of pulmonary surfactant can be considered as a potential therapy for ARDS patients.

**Table 1:** The mechanism of action for surfactant in ARDS

The mechanism of action for surfactant	
The capacity to maintain lower alveolar tension and stability of alveolar volume	
Promotion of gas exchange and distribution	
Anti-action of edema in alveoli and interstitium	
Modulation of systemic inflammatory reactions in ARDS	
Reduction of local mechanical forces in ARDS	

Currently, pulmonary surfactant is regarded as standard treatment for children with acute respiratory failure [4, 5]. Considering the impact of pulmonary surfactant on adult ARDS patients, a number of studies have explored the clinical benefits of administering pulmonary surfactant to adult patients with ARDS. However, individual studies have yielded inconsistent or conflicting findings. To shed light on these contradictory results and more precisely evaluate pulmonary surfactant on adult ARDS patients, we performed a meta-analysis of randomized controlled trials (RCTs) of pulmonary surfactant administration therapy on adult ARDS patients.

## Methods

### Data sources and searches

Databases (PubMed,EMBASE, Medline, Cochrane database, Elsevier, Web of Science and ClinicalTrials.gov) were searched until December 2017. Searches strategies were used with medical key words:<‘adult respiratory distress syndrome’, ‘acute respiratory distress syndrome’, or ‘ARDS’>; <‘pulmonary surfactant’ or‘lung surfactant’>; and < ‘adult’>. We conducted manual searching techniques to identify appropriate studies and applied no language restrictions. Randomized controlled clinical trials using adult participants (older than 18 years) were included in this meta analysis.

### Data extraction and study selection

Two reviewers (S-S.M., W.C.) independently screened and extracted titles, abstracts, and citations to evaluate each study and any disagreements were resolved by third reviewer (F-M.G.). The investigators selected and determined the enrolled studies depending on the inclusion and exclusion criteria.

### Inclusion and exclusion criteria

Trials with following features were included: 1) Type of study: Randomized controlled clinical trials; 2) Population: Adult patients (older than 18 years) who were diagnosed with acute respiratory distress syndrome; 3) Intervention: pulmonary surfactant administration; 4) Control: ARDS standard treatment; 5) The following outcomes were included. a) Primary outcomes: mortality at short term (7–10-day), mid-term (28–30-day) and long term (90–180-day); b) Secondary outcomes: PaO_2_/FiO_2_ (mmHg).

Exclusion criteria were as follows: 1) The age of participants were lower than 18 years old; 2) Trial with insufficient information; 3) The study was a review, case report, letter, or other type of publication and animal trial; 4) The study was non-randomized controlled trial; 5) the study did not include mortality or PaO_2_/FiO_2_ data; and 6) the full text was unavailable.

### Quality assessment

According to the Cochrane Handbook, random sequence generation, allocation concealment, blinding of participants and personnel, blinding of outcome assessment, incomplete outcome data and selective reporting were assessed to research the internal validity of included trials.

### Assessment of bias risk

Trial sequential analysis (TSA; TSA software version 0.9 Beta; Copenhagen Trial Unit, Copenhagen, Denmark) was applied to help to clarify whether additional trials are needed in the cumulative meta-analysis. TSA also controls the risks of type I and type II errors for meta-analysis [6, 7].

Grading of Recommendations Assessment, Development, and Evaluation methodology (GRADE) pro Guideline Development Tool were conducted to evaluate design, quality, consistency, precision, directness and possible publication bias of the included trials. GRADE was assessed in three levels (high, moderate, low, and very low).

### Data synthesis and analysis

We conducted a meta-analysis on the effect of pulmonary surfactant to adult ARDS patients using the methods recommended by the Cochrane Collaboration software RevMan 5.3 (The Nordic Cochrane Centre, Rigshospitalet, Copenhagen, Denmark). Risk ratio (RR) was reported with 95% confidence interval (CI) for the dichotomous data and weighted mean differences(MD) with 95% CIs for the continuous data. A Z-test was performed to statistically evaluate the treatment effects in different groups (13). We measured and quantified the statistical heterogeneity and inconsistency by the Mantel-Haenszel (M-H) chi-square test and the I^2^ test in RevMan 5.3 [8]. The statistically significant heterogeneity was evaluated as *p* < 0.10 with the M-H chi-square test. In addition, we assess I^2^ index as heterogeneity. Higgins and colleagues proposed 25, 50 and 75% of I^2^ values would indicate low, medium and high heterogeneity, respectively [8]. A fixed-effect model was used unless there was significant heterogeneity, in which case we applied a random effects model. In cases of obvious heterogeneity (p < 0.10 with M-H test; I^2^ > 50%), the meta-analysis employed the random-effects model; otherwise, the meta-analysis used the fixed-effects model.

### Subgroup meta-analysis

A subgroup meta-analysis was performed to determine the effect of surfactant administration on outcomes of acute respiratory distress syndrome patients. The primary outcome of the surfactant effect was selected as mortality. Mortality of ARDS patients were classified into short term mortality (7–10-day), mid-term mortality(28–30-day) and long term mortality (90–180-day). Thus, we performed three subgroups meta-analysis of different terms of ARDS mortality. Acute physiology and chronic health evaluation II (APACHE II) is positive correlation of illness severity. The patients of mean APACHE II > 15 were regarded as more severe ARDS and also investigated to analysis 28–30-day mortality.

### Sensitivity analyses

Sensitivity analyses were used to assess the impact of study quality issues on the overall effect estimate and the effect size of all identified trials when neglecting heterogeneity and publication status. Sensitivity analyses were conducted by STATA11.0 (Stata Corporation, College Station, TX, USA). A statistical test for funnel plot asymmetry was used to investigate the publication bias. Egger’s test and Begger ‘s inspection were also used to assess bias of meta-analysis conducted by STATA11.0 (Stata Corporation).

## Results

### Literature search

**Fig. 1:**
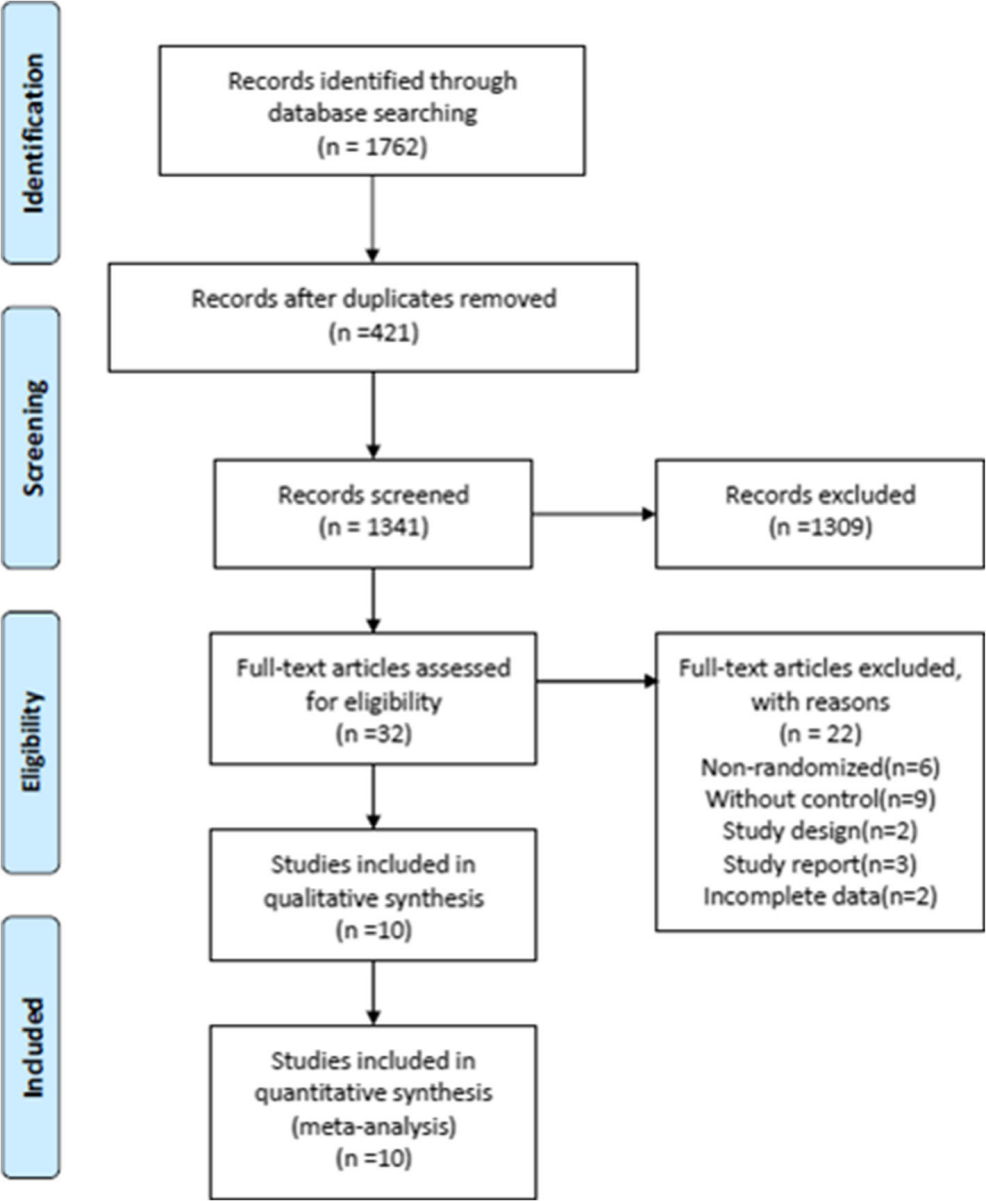
Flow diagram of the study selection

The process of study selection was presented as flow diagram in Fig. 1. We initially identified 1762 papers and excluded 421 duplicates references and 1730 references after screening the titles and abstracts for the terms “surfactant”, “acute respiratory distress syndrome” and “randomized control trial”. We assessed 32 articles for eligibility and excluded 6 non-randomized references, 9 studies without control, 3 reports, 2 inconformity study design references and 2 incomplete data references. Finally, 10 were included in this meta-analysis [9–18]. The RCT by Spragg 2004 [13] was conducted including results from both a North American trial (NA) and a European–South African trial (ES). The data from the two trials in this manuscript were assessed independently. Thus, 11 RCTs were enrolled in our meta-analysis. Eleven trials included 3038 patients. 1545 ARDS patients who received surfactant administration were regarded as experiment group, whereas control group (only received ARDS general therapy). The baseline characteristics of the included RCTs were shown in Table 2.

**Table 2:** Baseline characteristics of the RCTs

study,year	Numbers of patients	Delivery method	Type of surfactant	Surfactant dosing(Total)	Treatment duration	Predisposing event
Weg 1994 [9]	51(S,34;C,17)	Aerosolized	exosurf(no surfactant protein)	21.9 or 43.5 mg DPPC/Kg/day	120 h	sepsis
Anzueto 1996 [10]	725(S,364;C,361)	Aerosolized	exosurf(no surfactant protein)	112 mg DPPC/Kg/day	5 days	sepsis
Gregory 1997 [11]	59(S,43;C,16)	Intratracheal	bovine lung extract(containing SP-B.C)	150 or 2100 or 3100 mg/kg	96 h	trauma,aspiration,transfusions,sepsis
Spragg 2003 [12]	40(S,27;C,13)	Intratracheal	Venticute(rSP-C-based surfactant)	11 or 20.5 ml/kg	24 h	burn,aspiration,sepsis,pneumonia,trauma,pancreatitis
Spragg 2004(ES) [13]	227(S,118;C,109)	Intratracheal	rSP-C-based surfactant	1 ml/kg	24 h	trauma,aspiration,transfusions,sepsis,burn,toxic injury
Spragg 2004(NA) [13]	221(S,106;C,115)	Intratracheal	rSP-C-based surfactant	1 ml/kg	24 h	trauma,aspiration,transfusions,sepsis,burn,toxic injury
Tsangaris 2007 [14]	16(S,8;C,8)	Intratracheal	natural bovine surfactant	200 mg/kg	72 h	blunt chest trauma
Kesecioglu 2009 [15]	418(S,208;C,210)	Intratracheal	natural(porcine)(containing SP-B.C)	600 mg/kg	36 h	sepsis,trauma,aspiration,shock,pneumonia
Spragg 2011 [16]	843(S,419;C,424)	Intratracheal	rSP-C-based surfactant	1 ml/kg	96 h	aspiration,pneumonia
Lu 2010 [17]	20(S,10;C,10)	Intratracheal	HL-10(containing SP-B.C)	600 mg/kg	36 h	bronchopneumonia,aspiration pneumonia,lung contusion,sepsis
Willson 2015 [18]	308(S,151;C,157)	Intratracheal	Pneumasurf(containing SP-B.C)	30 mg per centimeter of height	12 h	viral,bacterial and aspiration pneumonia,et al
study,year		Initial APACHE II Score		Initial PaO2/FiO2	Enrolled hospitals	The limitation of enrolled study
Weg 1994 [9]		C:14.2(6.4) S1: 16.5(6.7) S2:15.7(6.6)		C:146.5(20.4) S1:124.2(11.8) S2:161.5(16.2)	20 tertiary care medical centers throughout the United States	Small sample
Anzueto 1996 [10]		Not available		C:140(64) S:145(82)	Medical–surgical intensive care units of 63 hospitals in nine countries	Complex causes of ARDS; Inadequate efficiency of surfactant reached the lungs; Incomplete surfactant preparation
Gregory 1997 [11]		Not available		C:128(71–286) S1:98(84–402); S2:124(40–234) S3: 133(77–401)	Five clinical centers: The Ohio State University Hospital (Columbus, OH); St. Louis University Health Sciences Center (St. Louis, MO); Harborview Medical Center (Seattle, WA);University of California, San Diego; and UCLA Medical Center(Los Angeles, CA)	Complex causes of ARDS; Inadequate efficiency of surfactant reached the lungs
Spragg 2003 [12]		C:10.9(1.1) S1: 10.2(1.2) S2:10.1(1.7)		C:133.6(8.9) S:113.9(8.3)	Hospitals in North America	Small sample; Inadequate efficiency of surfactant reached the lungs
Spragg 2004(ES) [13]		C:16.6(5.8) S:17.4(7.5)		C:136(39) S:137(40)	55 centers in Austria, Belgium, France, Germany, the Netherlands, South Africa, Spain, Switzerland, and the United Kingdom.	Narrow treatment window
Spragg 2004(NA) [13]		C:17.9(6.6) S:18.6(6.1)		C:130(39) S:132(40)	54 centers in Canada and the United States	Narrow treatment window
Tsangaris 2007 [14]		C:16(4) S:15(3)		C:103(14) S:100(20)	14-bed ICU in Greece	Small sample; a single and reduced surfactant dose
Kesecioglu 2009 [15]		C:25.2(7.3) S:25.7(8.2)		C:161.4(55.2) S:156.7(54.8)	67 medical centers in Austria, Belgium, Canada, Denmark, Finland, France, Germany, the Netherlands, Norway, Spain, Sweden, and the United Kingdom	Different confounding factors; Heterogeneous nature of ALI/ARDS population; Definition of ALI/ARDS
Spragg 2011 [16]		C:17.8(0.32) S:18(0.33)		C:124.1(1.32) S:123.8(1.3)	Intensive care units of 161 medical centers in 22 countries.	Complex causes of ARDS
Lu 2010 [17]		Not available		C:200(63) S:201(64)	multidisciplinary ICU of La Pitié-Salpêtrière Hospital, University Pierre et Marie Curie, Paris, France,	Small sample
Willson 2015 [18]		C:60(28) S:63(31)		C1:147, S1:135 C2:143, S2:136	Intensive care units of 34 medical centers in 6 countries	Complex causes of ARDS

*C* control, *S* surfactant

### Random errors

**Fig. 2:**
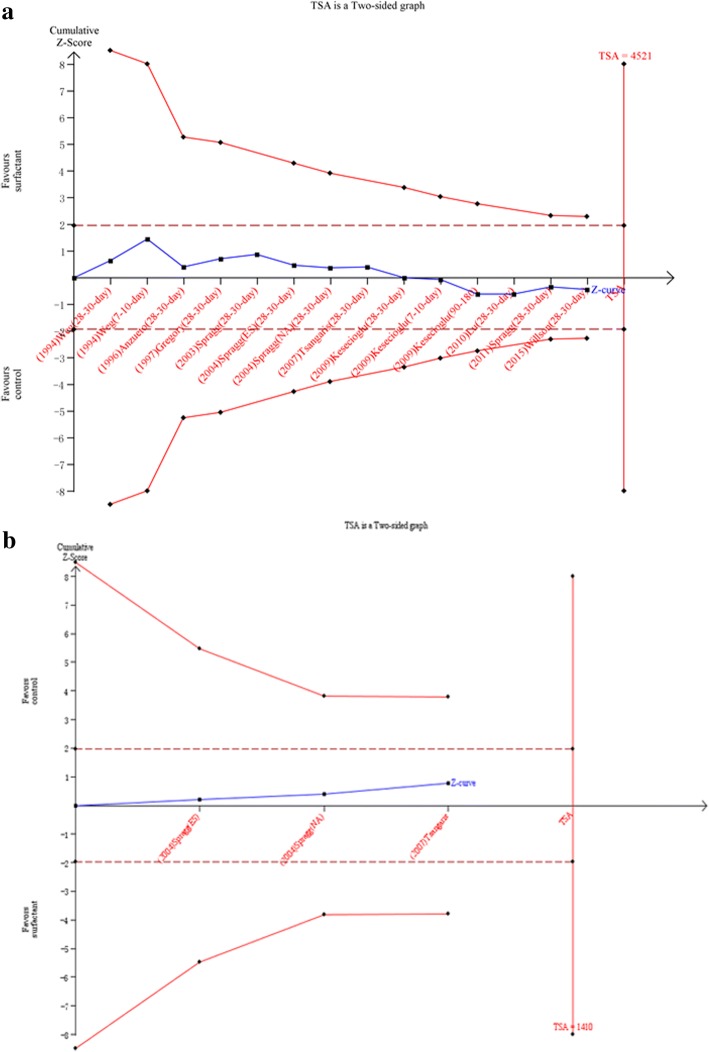
Trial sequential analysis for outcomes in adult ARDS patients after surfactant therapy. **a** mortality of ARDS. **b** value of PaO2/FiO2(Fig. 2b)

Trial sequential analysis was calculated for mortality of ARDS patients and the value of PaO2/FiO2 after surfactant therapy. TSA was calculated with α = 0.05 and β = 0.20 (power 80%) and a required diversity-adjusted information size based on the intervention effect suggested by the included trials using fixed-effects models. The cumulated Z-curve (blue) doesn’t crosses the traditional boundary and trial sequential monitoring boundary, indicating that lack of reliable and conclusive evidence for beneficial effects of pulmonary surfactant for both mortality (Fig. 2a) and PaO2/FiO2 outcome (Fig. 2b). There is insufficient information to assess the effect of surfactant for ARDS patients.

### Surfactant administration can not improve mortality of acute respiratory distress syndrome patients

**Fig. 3:**
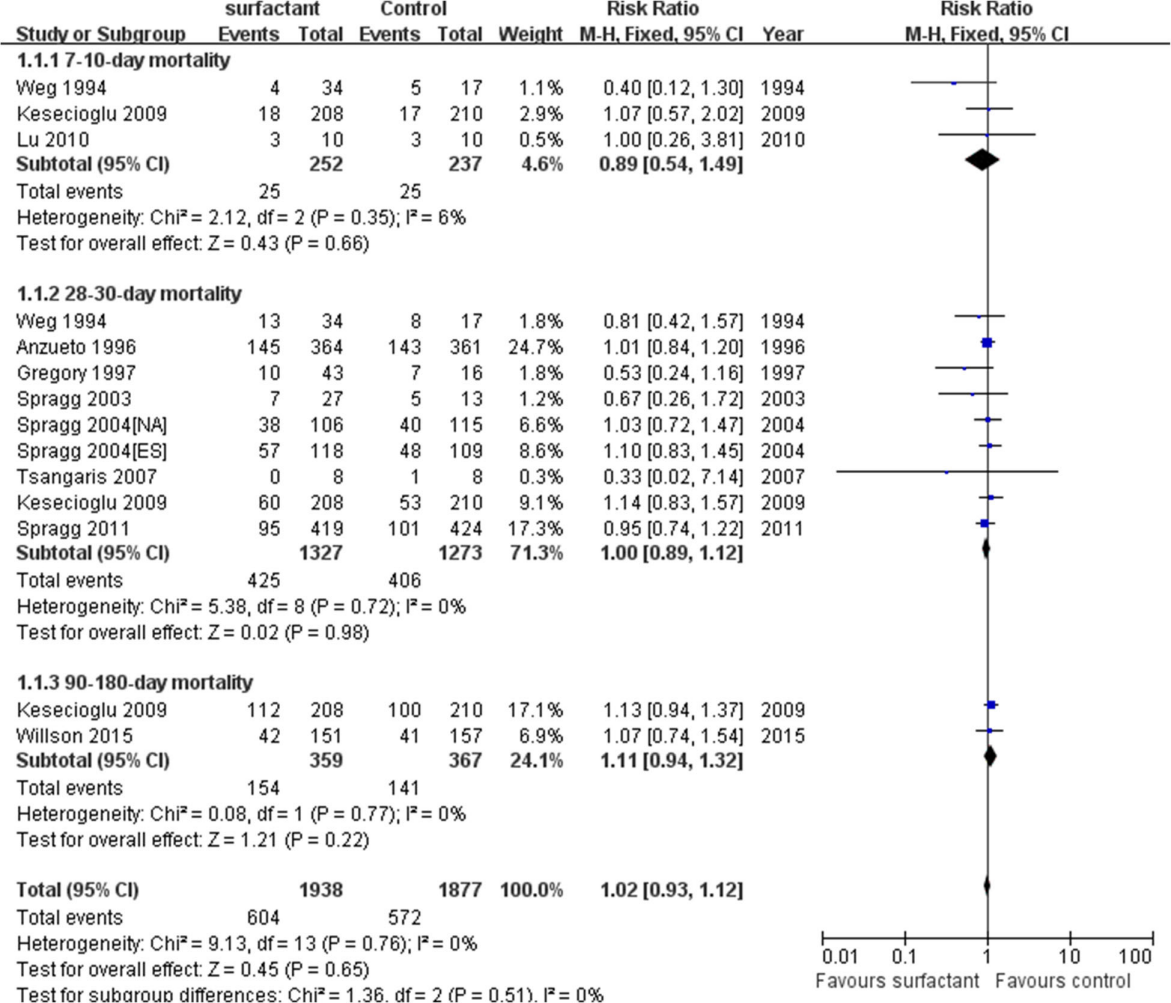
Forest plots of subgroup analyses on the effect of surfactant based on mortality. CI Confidence interval, M-H Mantel-Haenszel

**Fig. 4:**
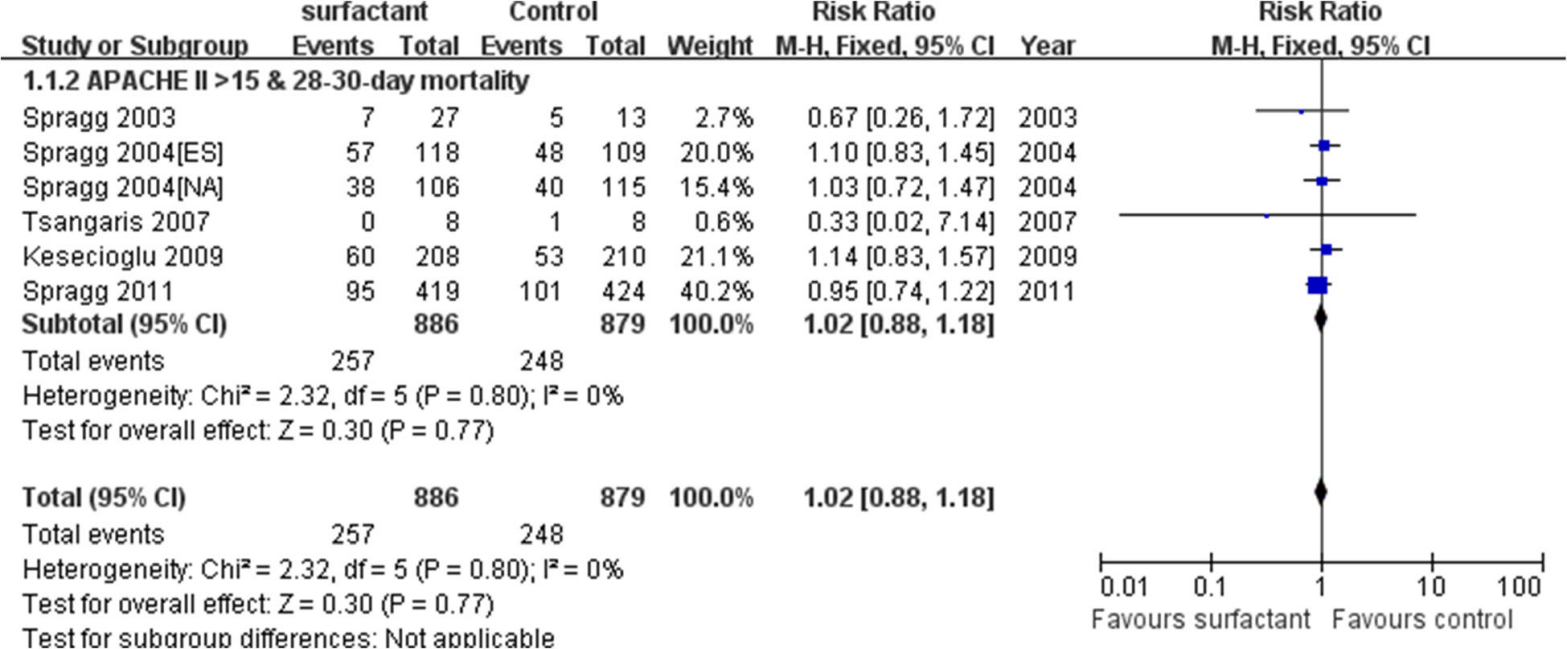
Forest plots of analyses on the effect of surfactant based on 28–30-day mortality(APACHE II > 15). CI Confidence interval, M-H Mantel-Haenszel

Among the included studies, eleven RCTs reported the mortality and were included in the primary analysis. We detected no evidence of a publication bias after a funnel plot analysis (Additional file 1: Figure S1a). Egger’s test and Begger’s inspection (*p* > 0.01) also implied no publication bias in mortality. In Fig. 3, there was not statistically insignificant heterogeneity (*p* = 0.76) and medium heterogeneity (I^2^ = 0%) among all mortality in our meta-analysis. Test for overall effect of mortality between surfactant group and control group has no differences[RR(95%CI) =1.02(0.93–1.12), *p* = 0.65]. Moreover, a subgroup analysis showed that 7–10-day, 28–30-day and 90–180-day mortality between surfactant group and control group also showed no statistical significance. In analysis of 28–30-day mortality, test for overall effect of 28–30-day mortality (APACHE II > 15) between surfactant group and control group has also no differences [RR(95%CI) =1.02(0.88–1.18), *p* = 0.77](Fig. 4). Three studies included 7–10-mortality (RR(95%CI)=)0.89(0.54–1.49), *p* = 0.66), nine studies included 28–30-mortality(RR(95%CI) =1.00(0.89–1.12), *p* = 0.98) and two studies included 90–180-day mortality(RR(95%CI) =1.11(0.94–1.32), *p* = 0.22) (Fig. 3). Sensitive analysis of comparison between surfactant and placebo group showed the result is stable (Additional file 1: Figure S2). Overall, we concluded that surfactant administration could not improve mortality of adult acute respiratory distress syndrome patients.

### Surfactant administration has no significant improvement in PaO2/FiO2 ratio of acute respiratory distress syndrome patients

**Fig. 5:**
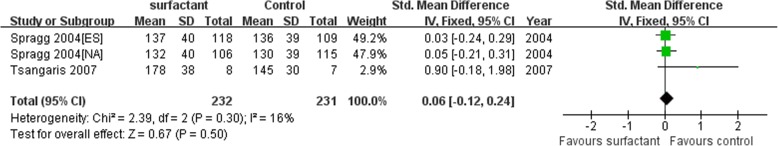
Forest plots of the effect of surfactant based on PaO2/FiO2. CI Confidence interval, M-H Mantel-Haenszel

We further made the meta-analysis of the result of PaO2/FiO2 ratio and three RCTs were included. In Fig. 5, there was not statistically insignificant heterogeneity (*p* = 0.3) and medium heterogeneity (I^2^ = 16%) among PaO2/FiO2 ratio. Test for overall effect of PaO2/FiO2 ratio between surfactant group and control group had no obvious differences[MD(95%CI) =0.06(− 0.12–0.24), *p* = 0.5]. Taken together, these suggested that surfactant administration could not improve PaO2/FiO2 ratio of adult acute respiratory distress syndrome patients.

### Evaluation of publication bias

**Fig. 6:**
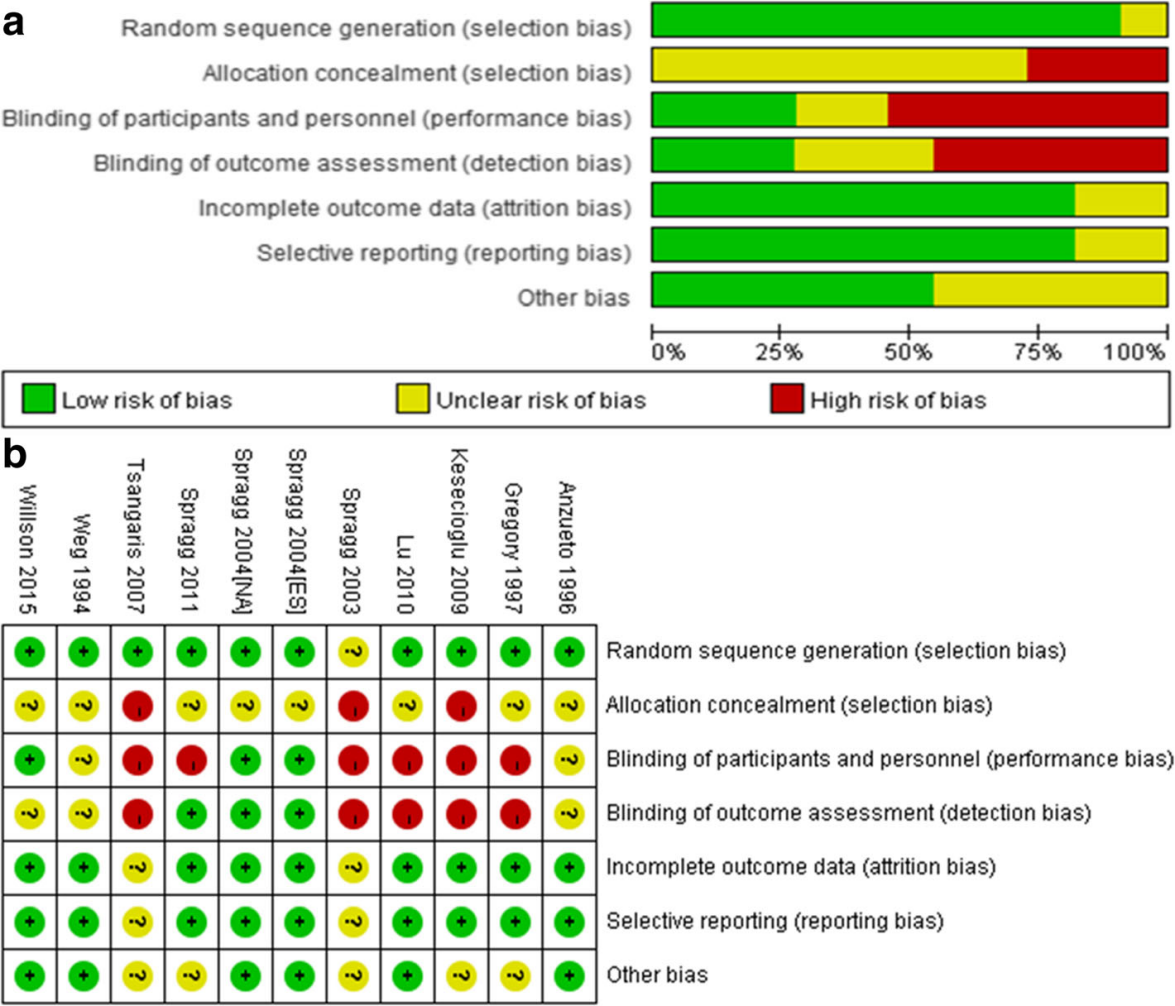
Risk bias analysis for enrolled studies. **a** Risk of bias graph: review authors’ judgments about each risk of bias item presented as percentages across all included studies. **b** Risk of bias summary: review authors’ judgments about each risk of bias item for each included study

We assessed each enrolled RCT by the mode of randomization, allocation concealment, level of blinding, incomplete outcome data, selective reporting and other bias (Fig. 6).

### Summary of evidence according to grade

RCTs are often rated high on the GRADE scale. Variable risks of bias in all the trials lead us to downgrade the quality of the evidence. Allocation concealment was not reported totally, and the sample sizes were all small. Our application of GRADE methodology led us to conclude that the accumulated evidence is of low quality for mortality and PaO2/FiO2 ratio. For a GRADE profile see Additional file 1: Table S1.

## Discussion

Many researches have exhibited it plays an important role in pediatric patients [4], and it seems sensible that surfactant would be a useful therapy in adult patients. Thus,our meta-analysis selected 11 randomized controlled trials. It demonstrated that there was no overall improvement in mortality (RR 1.02; 95% CI 0.93, 1.12). Furthermore, subgroup analysis of short, middle and long term mortality did not demonstrate improved outcomes. In three of the studies we were not able to assess the impact of surfactant on oxygenation (PaO_2_/FiO_2_ ratio). There was no improved oxygenation after surfactant administration (MD 0.06; 95% CI -0.12, 0.24). APACHE II > 15 was not considered as a factor effecting 28–30-day mortality with surfactant administration (RR 1.02; 95%CI 0.88, 1.18).

The trials we selected were all randomized controlled trials. Unlike the most recent published meta–analysis, we updated the meta-analysis with Lu [16] and Willson [17] research. Depending on clinical practice, we defined mortality as primary outcome, and PaO_2_/FiO_2_ ratio as secondary outcome. We firstly classified mortality as three different subgroups, short term mortality(7–10-day), mid-term mortality(28–30-day) and long term mortality(90–180-day). Moreover, we applied trial sequential analysis to help to clarify whether additional trials are needed in the cumulative meta-analysis and control the risks of type I and type II errors. We used GRADE to evaluate design, quality, consistency, precision, directness and possible publication bias of the included trials and levels of trials.

Unfortunately, the quality of the studies varied in our meta-analysis. The sample sizes were all small. Allocation concealment was not reported totally, and three trials did not have unequivocal blinding method. It is possible that we may have missed some important information and get an inadequate result.

Adult ARDS patient usually exhibit the surfactant change of amount and function. Although, surfactant is useful in children patients and has a clear effect, there are inadequate evidence of doses and administration methods due to the change of surfactant ingredients in adult patients. Pediatric patients usually have etiology of surfactant lack and meconium aspiration, which is unlike in adult patients of trauma, aspiration, transfusions, sepsis, burn and toxic injury etiology. Surfactant has been researched and regarded as immune regulator in patients. However, children’ immune characteristics are not same as adult. Recommended dose of surfactant to children patients is 100–200 mg/kg, and higher dose has not obvious effect. However, the doses to adult patients are not clear with various doses. Intratracheal administration with mechanical ventilation is a better method for surfactant administration [19]. Exogenous surfactant therapy has many administration methods. Thus, the reasons we demonstrated above give surfactant diffident effects to children and adult.

We further discovered that there was no improved oxygenation after surfactant supply. However, Lu et al. [16] reported increased lung aeration relative to placebo on CT scan when instillation was accompanied by a recruitment maneuver, increasing tidal volume to 12 ml/Kg PBW and PEEP by 5 cm H_2_O for 30 min after instillation. Recruitment maneuver may have transitory effect. Adult ARDS usually is characterized by loss of pulmonary endothelium and epithelium cells, sophisticated etiology, and disordered immune system; simple surfactant supply was not enough for adult ARDS patients. ARDS patients usually die of multi-organ system failure from their underlying disease process (for example sepsis) rather than from respiratory failure.

Although ARDS patients have deficiency of surfactant, the mechanisms of ARDS are complex. Surfactant administration may help improve the ARDS, but it is simply not sufficient for changing the outcome of adult ARDS patients. Varied factors including causes, severity, immune responses of patients and medical level of doctors influenced the results.

There were some limitations in our meta-analysis. Firstly, we applied different ingredients of surfactant. Details can be seen in Table 1. SP-A, SP-B, SP-C, and SP-D surfactant proteins have been previously identified. SP-B and SP-C are hydrophobic proteins that enhance the lowering of surface tension, and SP-A and SP-D are hydrophilic proteins whose role appears to center around host defense [20]. It is possible that the presence or absence of these proteins could change the effectiveness of therapy. Secondly, the different treatment duration used may have resulted in varying effects. Different treatment duration may have different pesticide effect and pharmacokinetics. Thirdly, different ventilation strategies were used resulting in different distribution concentration. High volume strategy of mechanical ventilation could facilitate surfactant distribution. In future studies, it would be interesting to explore the detailed mechanisms and relationships between surfactant distribution and different mechanical ventilation strategies.

## Conclusions

We found in our meta-analysis that administration of surfactant was not associated with improved mortality of adult ARDS patients. Surfactant instillation has no effects of oxygenation (PaO2/FiO2 ratio) improvement. Further RCTs of surfactant administration should be performed to explore the effect of surfactant to adult ARDS patients.

## Additional file

Additional file 1: **Figure S1.** Analysis of funnel plot for mortality outcomes in adult ARDS patients after surfactant therapy. **Figure S2.** Sensitive analysis for mortality outcomes of adult ARDS patients with surfactant therapy. **Table S1.** GRADE profile for quality assessment of evidence. (DOCX 148 kb)

## Abbreviations

APACHE II: Acute physiology and chronic health evaluation
ARDS: Acute respiratory distress syndrome
CI: Confidence interval
GRADE: Grading of Recommendations Assessment Development, and Evaluation methodology
RCT: Randomized controlled trial
RR: Risk ratio
TSA: Trial sequential analysis
