# Supplementary material for: Effect of surfactant administration on outcomes of adult patients in acute respiratory distress syndrome: a meta-analysis of randomized controlled trials
Source: BMC Pulm Med. 2019 Jan 9;19:9. doi: 10.1186/s12890-018-0761-y (PMC6325713; doi:10.1186/s12890-018-0761-y)
Supplement: Supplementary file 1 — Figure S1. Analysis of funnel plot for mortality outcomes in adult ARDS patients after surfactant therapy. Figure S2. Sensitive analysis for mortality outcomes of adult ARDS patients with surfactant therapy. Table S1. GRADE profile for quality assessment of evidence. (DOCX 148 kb) [file 12890_2018_761_MOESM1_ESM.docx]

***Online data supplement***

**Table S1** GRADE profile for quality assessment of evidence

| **Quality assessment** | | | | | | | **No of patients** | | **Effect** | | **Quality** | **Importance** |
| --- | --- | --- | --- | --- | --- | --- | --- | --- | --- | --- | --- | --- |
|  |  |  |  |  |  |  |  |  |  |  |  |  |
| **No of studies** | **Design** | **Risk of bias** | **Inconsistency** | **Indirectness** | **Imprecision** | **Other considerations** | **Surfactant** | **Placebo** | **Relative (95% CI)** | **Absolute** |  |  |
| **mortality (follow-up 0.5-5 days; assessed with: follow up)** | | | | | | | | | | | | |
| 11 | randomized trials | serious^1^ | no serious inconsistency | no serious indirectness | serious^2^ | none | 604/1938 (31.2%) | 572/1877 (30.5%) | OR 1.02 (0.93 to 1.12) | 6 more per 1000 (from 21 fewer to 37 more) | ⊕⊕OO LOW | CRITICAL |
|  |  |  |  |  |  |  |  | 32.4% |  | 6 more per 1000 (from 23 fewer to 39 more) |  |  |
| **PaO2/FiO2 ratio (follow-up 24-72 hours)** | | | | | | | | | | | | |
| 3 | randomized trials | very serious^3^ | no serious inconsistency | no serious indirectness | serious^4^ | none | 232 | 231 | - | SMD 0.06 higher (0.12 to 0.24higher) | ⊕OOO VERY LOW | IMPORTANT |

^1^  Allocation concealment were not reported totally.
^2, 4^ The sample sizes were all small.

^3^ Allocation concealment was not reported totally, and two trials did not have unequivocal blinding method.


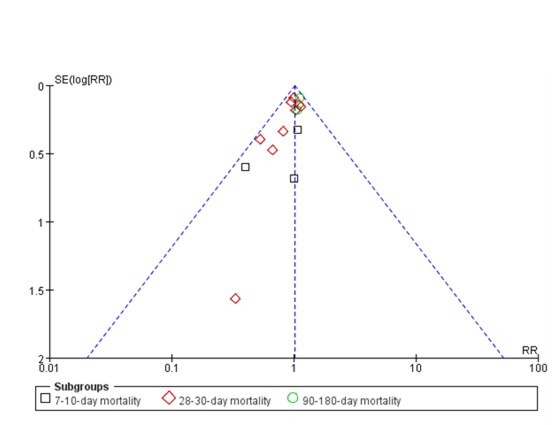


**Fig S1** Analysis of funnel plot for mortality outcomes in adult ARDS patients after surfactant therapy.


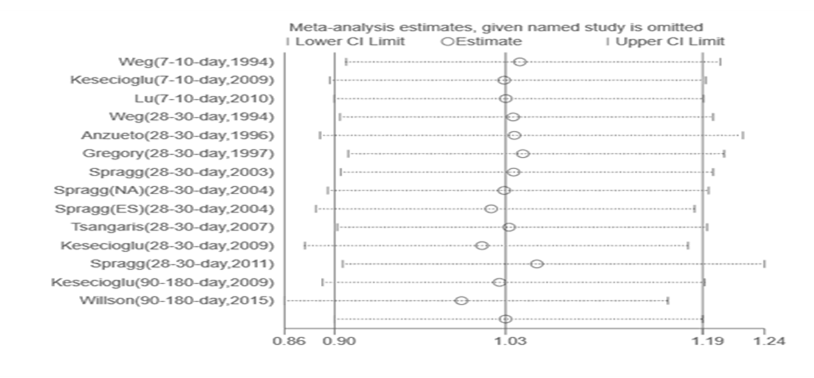


**Fig S2** Sensitive analysis for mortality outcomes of adult ARDS patients with surfactant therapy.
